# Supplementary material for: Deep Infiltrating Endometriosis and Adenomyosis: Implications on Pregnancy and Outcome
Source: J Clin Med. 2021 Dec 29;11(1):157. doi: 10.3390/jcm11010157 (PMC8745136; doi:10.3390/jcm11010157)
Supplement: Supplementary file 1 [file jcm-11-00157-s001.zip › jcm-1482735-supplementary.pdf]

Supplementary data:

Table S1:

**PRISMA 2009 Checklist**			
**Section/topic**	**#**	**Checklist item**	**Reported in chapter**
**TITLE**			
Title	1	Identify the report as a systematic review, meta-analysis, or both.	Systematic review
**ABSTRACT**			
Structured summary	2	Provide a structured summary including, as applicable: background; objectives; data sources; study eligibility criteria, participants, and interventions; study appraisal and synthesis methods; results; limitations; conclusions and implications of key findings; systematic review registration number.	Abstract
**INTRODUCTION**			
Rationale	3	Describe the rationale for the review in the context of what is already known.	Introduction
Objectives	4	Provide an explicit statement of questions being addressed with reference to participants, interventions, comparisons, outcomes, and study design (PICOS).	Material and Methods
**METHODS**			
Protocol and registration	5	Indicate if a review protocol exists, if and where it can be accessed (e.g., Web address), and, if available, provide registration information including registration number.	-
Eligibility criteria	6	Specify study characteristics (e.g., PICOS, length of follow-up) and report characteristics (e.g., years considered, language, publication status) used as criteria for eligibility, giving rationale.	Material and Methods
Information sources	7	Describe all information sources (e.g., databases with dates of coverage, contact with study authors to identify additional studies) in the search and date last searched.	Material and Methods
Search	8	Present full electronic search strategy for at least one database, including any limits used, such that it could be repeated.	Material and Methods, Supplement B
Study selection	9	State the process for selecting studies (i.e., screening, eligibility, included in systematic review, and, if applicable, included in the meta-analysis).	Material and Methods Supplement B
Data collection process	10	Describe method of data extraction from reports (e. g., piloted forms, independently, in duplicate) and any processes for obtaining and confirming data from investigators.	Material and Mehods
Data items	11	List and define all variables for which data were sought (e.g., PICOS, funding sources) and any assumptions and	Material and Methods

PRISMA 2009 Checklist			
**Section/topic**	**#**	**Checklist item**	**Reported in chapter**
		simplifications made.	
Risk of bias in individual studies	12	Describe methods used for assessing risk of bias of individual studies (including specification of whether this was done at the study or outcome level), and how this information is to be used in any data synthesis.	Limitations Supplement B
Summary measures	13	State the principal summary measures (e.g., risk ratio, difference in means).	-
Synthesis of results	14	Describe the methods of handling data and combining results of studies, if done, including measures of consistency (e.g., I^2) for each meta-analysis.	-
Risk of bias across studies	15	Specify any assessment of risk of bias that may affect the cumulative evidence (e.g., publication bias, selective reporting within studies).	-
Additional analyses	16	Describe methods of additional analyses (e.g., sensitivity or subgroup analyses, meta-regression), if done, indicating which were pre-specified.	-
**RESULTS**			
Study selection	17	Give numbers of studies screened, assessed for eligibility, and included in the review, with reasons for exclusions at each stage, ideally with a flow diagram. .	Results
Study characteristics	18	For each study, present characteristics for which data were extracted (e.g., study size, PICOS, follow-up period) and provide the citations.	-
Risk of bias within studies	19	Present data on risk of bias of each study and, if available, any outcome level assessment (see item 12).	Supplement C
Results of individual studies	20	For all outcomes considered (benefits or harms), present, for each study: (a) simple summary data for each intervention group (b) effect estimates and confidence intervals, ideally with a forest plot.	Results
Synthesis of results	21	Present results of each meta-analysis done, including confidence intervals and measures of consistency.	-
Risk of bias across studies	22	Present results of any assessment of risk of bias across studies (see Item 15).	-
Additional analysis	23	Give results of additional analyses, if done (e.g., sensitivity or subgroup analyses, meta-regression.	-
**DISCUSSION**			
Summary of evidence	24	Summarize the main findings including the strength of evidence for each main outcome; consider their relevance to key groups (e.g., healthcare providers, users, and policy makers).	Discussion

PRISMA 2009 Checklist			
**Section/topic**	**#**	**Checklist item**	**Reported in chapter**
Limitations	25	Discuss limitations at study and outcome level (e.g., risk of bias), and at review-level (e.g., incomplete retrieval of identified research, reporting bias).	Limitations
Conclusions	26	Provide a general interpretation of the results in the context of other evidence, and implications for future research.	Conclusion
**FUNDING**			
Funding	27	Describe sources of funding for the systematic review and other support (e.g., supply of data); role of funders for the systematic review.	-

Figure S1: Search Terms

PubMed search term (10.03.2020)"adenomyosis"[All Fields] OR "deep infiltrating endometriosis"[All Fields] OR "deep endometriosis"[All Fields] OR "rectovaginal endometriosis"[All Fields]) AND ("delivery, obstetric"[MeSH] OR "pregnancy complications"[MeSH] OR "infant, newborn"[MeSH]) AND ("humans"[MeSH] AND (English[lang] OR French[lang] OR German[lang] OR Spanish[lang])

Scopus search term (10.03.2020)

TITLE-ABS-KEY ("adenomyosis" OR "deep infiltrating endometriosis" OR "deep endometriosis" OR "rectovaginal endometriosis") AND TITLE-ABS-KEY ("prematurity" OR "premature birth" OR "cesarean section" OR "mode of delivery" OR "pregnancy complications" OR "pregnancy outcome" OR "prenatal care" OR "obstetrics") AND (EXCLUDE (DOCTYPE , "ed") OR EXCLUDE (DOCTYPE , "le") OR EXCLUDE (DOCTYPE , "no") OR EXCLUDE (DOCTYPE , "cp") OR EXCLUDE (DOCTYPE , "er") OR EXCLUDE (DOCTYPE , "sh")) AND (LIMIT-TO (DOCTYPE , "ar") OR LIMIT-TO (DOCTYPE , "re")) AND (LIMIT-TO (EXACTKEYWORD , "Human")) AND (LIMIT-TO (LANGUAGE , "English") OR LIMIT-TO (LANGUAGE , "French") OR LIMIT-TO (LANGUAGE , "Spanish") OR LIMIT-TO (LANGUAGE , "German"))

Web of Science search term (10.03.2020)

TS=(adenomyosis OR deep infiltrating endometriosis OR deep endometriosis OR rectovaginal endometriosis) AND TS=(prematurity OR premature birth OR cesarean section OR mode of delivery OR pregnancy complications OR pregnancy outcome OR prenatal care OR obstetrics)
Refined by: DOCUMENT TYPES: (ARTICLE OR REVIEW) AND LANGUAGES: (ENGLISH OR SPANISH OR FRENCH OR GERMAN)
Indexes=SCI-EXPANDED, SSCI, A&HCI, CPCI-S, CPCI-SSH, BKCI-S, BKCI-SSH, ESCI, CCR-EXPANDED, IC Timespan=All years

Table S2:

Newcastle-Ottawa Quality Assessment Scale for Cohort Studies								
Cohort studies<br><br>Note: *A study can be awarded a maximum of one star for each numbered item within the Selection (S) and Outcome (O) categories. A maximum of two stars can be given for Comparability (C).*			AM					DIE
			Porpora 2020	Yamaguchi [illegible]	Harada 2019	Sharma 2019	Shin 2018	Uccella 2019
**S**	1) Representativeness of the exposed cohort	a) truly representative of the average *patient with EM* in the community	-			-		-
		b) somewhat representative of the average *patient with EM* in the community		-	-		-	
		c) selected group of users e.g. nurses, volunteers	-	-	-	-	-	-
		d) no description of the derivation of the cohort	-	-	-	-	-	-
	2) Selection of the non-exposed cohort	a) drawn from the same community as the exposed cohort						
		b) drawn from a different source	-	-	-	-	-	-
		c) no description of the derivation of the non-exposed cohort	-	-	-	-	-	-
	3) Ascertainment of exposure	a) secure record (e.g., surgical records)		-	-			
		b) structured interview	-	-	-	-	-	-
		c) written self-report	-	+	+	-	-	-
		d) no description	-	-	-	-	-	-
	4) Demonstration that outcome of interest was not present at start of study	a) yes						
		b) no	-	-	-	-	-	-
**C**	1) Comparability of cohorts on the basis of the design or analysis	a) study controls for *previous cesarean section and parity*		-	-	-	-	
		b) study controls for *multiple pregnancies and IVF/ICSI*	-			-		-
**O**	1) Assessment of outcome	a) independent blind assessment	-	-	-	-	-	-
		b) record linkage						
		c) self-report	-	-	-	-	-	-
		d) no description	-	-	-	-	-	-

Newcastle-Ottawa Quality Assessment Scale for Cohort Studies								
Cohort studies<br><br>*Note: A study can be awarded a maximum of one star for each numbered item within the Selection (S) and Outcome (O) categories. A maximum of two stars can be given for Comparability (C).*			AM					DIE
			Porpora 2020	Yamaguchi	Harada 2019	Sharma 2019	Shin 2018	Uccella 2019
	2) Was follow-up long enough for outcomes to occur	a) yes (follow-up until discharge from birth-clinic after delivery)						
		b) no	-	-	-	-	-	-
	3) Adequacy of follow up of cohorts	a) complete follow up - all subjects accounted for	-	-	-		-	-
		b) subjects lost to follow up unlikely to introduce bias - small number lost (<15% lost to follow up, or description provided of those lost)	-			-	-	-
		c) follow up rate >15% and no description of those lost	-	-	-	-	+	-
		d) no statement	+	-	-	-	-	+
		**Summe**	7/9	7/9	7/9	7/9	7/9	7/9

Newcastle-Ottawa Quality Assessment Scale for Case Control Studies											
Case control studies<br><br>*Note: A study can be awarded a maximum of one star for each numbered item within the Selection (S) and Exposure (E) categories. A maximum of two stars can be given for Comparability (C).*			AM				DIE				
			Hashimoto	Hasdemir	Mochimaru	Juang	Scala	Nirgianakis	Mannini 2017	Exacoustos	Baggio 2015
**S**	1) Is the case definition adequate?	a) yes, with independent validation						-			-
		b) yes, e.g., record linkage or based on self-reports	-	-	-	-	-	+	-	-	+
		c) no description	-	-	-	-	-	-	-	-	-
	2) Representativeness of the cases	a) consecutive or obviously representative series of cases					-				
		b) potential for selection biases or not stated	-	-	-	-	+	-	-	-	-
	3) Selection of Controls	a) community controls	-	-				-			-
		b) hospital controls	+	+	-	-	-	+	-	-	+

Newcastle-Ottawa Quality Assessment Scale for Case Control Studies

Case control studies			AM				DIE				
Note: A study can be awarded a maximum of one star for each numbered item within the Selection (S) and Exposure (E) categories. A maximum of two stars can be given for Comparability (C).			Hashimoto	Hasdemir	Mochimaru	Juang	Scala	Nirgianakis	Mannini 2017	Exacoustos	Baggio 2015
		c) no description	-	-	-	-	-	-	-	-	-
	4) Definition of Controls	a) no history of disease (endpoint)		-							
		b) no description of source	-	+	-	-	-	-	-	-	-
**C**	1) Comparability of cases and controls on the basis of the design or analysis	a) study controls for *previous cesarean section or parity*	-	-	-	-	-		-	-	-
		b) study controls for *multiple pregnancies or IVF/ICSI*		-	-	-			-	-	-
**E**	1) Ascertainment of exposure	a) secure record (e.g., surgical records)		-							
		b) structured interview where blind to case/control status	-	-	-	-	-	-	-	-	-
		c) interview not blinded to case/control status	-	-	-	-	-	-	-	-	-
		d) written self-report or medical record only	-	+	-	-	-	-	-	-	-
		e) no description	-	-	-	-	-	-	-	-	-
	2) Same method of ascertainment for cases and controls	a) yes	-		-		-	-	-	-	-
		b) no	+	-	+	-	+	+	+	+	+
	3) Non-Response rate	a) same rate for both groups	-	-			-	-	-	-	-
		b) non respondents described	-	-	-	-	-	-	-	-	-
		c) rate different and no designation	+	+	-	-	+	+	+	+	+
		**Summe**	5/9	3/9	6/9	7/9	5/9	5/9	5/9	5/9	3/9
